# A reliable workflow for improving nanoscale X-ray fluorescence tomographic analysis on nanoparticle-treated HeLa cells

**DOI:** 10.1093/mtomcs/mfac025

**Published:** 2022-06-25

**Authors:** Yanqi Luo, Tatjana Paunesku, Olga Antipova, Yuzi Liu, Nestor J Zaluzec, Zichao Di, Gayle Woloschak, Si Chen

**Affiliations:** X-ray Science Division, Advanced Photon Source, Argonne National Laboratory, Lemont, IL 60439, USA; Department of Radiation Oncology, Feinberg School of Medicine, Northwestern University, Chicago, IL 60611, USA; X-ray Science Division, Advanced Photon Source, Argonne National Laboratory, Lemont, IL 60439, USA; Center for Nanoscale Materials, Argonne National Laboratory, Lemont, IL 60439, USA; Photon Sciences Directorate, Argonne National Laboratory, Lemont, IL 60439, USA; Mathematics and Computer Science Division, Argonne National Laboratory, Lemont, IL 60439, USA; Department of Radiation Oncology, Feinberg School of Medicine, Northwestern University, Chicago, IL 60611, USA; X-ray Science Division, Advanced Photon Source, Argonne National Laboratory, Lemont, IL 60439, USA

**Keywords:** synchrotron based microscopy, Multiscale microscopy analysis, Focusedion beam, Nanoparticles and HeLa cells, Trace element mapping, X-ray fluorescence

## Abstract

Scanning X-ray fluorescence (XRF) tomography provides powerful characterization capabilities in evaluating elemental distribution and differentiating their inter- and intra-cellular interactions in a three-dimensional (3D) space. Scanning XRF tomography encounters practical challenges from the sample itself, where the range of rotation angles is limited by geometric constraints, involving sample substrates or nearby features either blocking or converging into the field of view. This study aims to develop a reliable and efficient workflow that can (1) expand the experimental window for nanoscale tomographic analysis of local areas of interest within a laterally extended specimen, and (2) bridge 3D analysis at micrometer and nanoscales on the same specimen. We demonstrate the workflow using a specimen of HeLa cells exposed to iron oxide core and titanium dioxide shell (Fe_3_O_4_/TiO_2_) nanocomposites. The workflow utilizes iterative and multiscale XRF data collection with intermediate sample processing by focused ion beam (FIB) sample preparation between measurements at different length scales. Initial assessment combined with precise sample manipulation via FIB allows direct removal of sample regions that are obstacles to both incident X-ray beam and outgoing XRF signals, which considerably improves the subsequent nanoscale tomography analysis. This multiscale analysis workflow has advanced bio-nanotechnology studies by providing deep insights into the interaction between nanocomposites and single cells at a subcellular level as well as statistical assessments from measuring a population of cells.

## Graphical Abstract

**Graphical Abstract:**
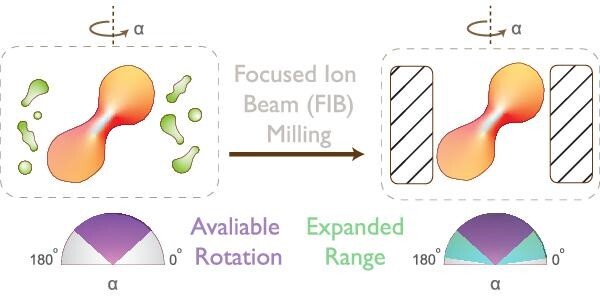
Focused ion beam (FIB) expanded angular coverage to improve scanning tomographic reconstruction

## Introduction

Bio-nanotechnology, particularly with the usage of nanoparticles, has led to promising results in cancer diagnosis and treatment.^1^ For example, paramagnetic Mn_8_Fe_4_-*co*-polystyrene nanobeads have the potential to be used as the exogenous contrast agent for T1–T2 magnetic resonance imaging,^2^ and Fe_3_O_4_/TiO_2_ nanocomposites could be introduced to enhance radiation effects in neuroblastoma treatment.^3^ Despite the great potential of bio-nanotechnology, gaining insights into the interactions between nanomaterials and subcellular structures is crucial prior to their wide applications *in vivo*. Questions such as nanomaterial behaviors within cancer and normal cells, complicated by the chemically heterogeneous nature of nanoparticles, are far from understood, especially at a subcellular level.

**Fig. 1:**
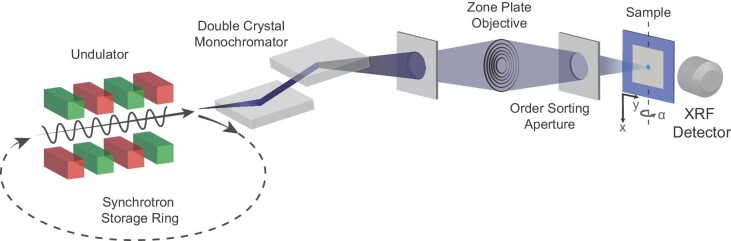
Schematic of the synchrotron-based X-ray fluorescence microscopy (XFM) setup.

Synchrotron-based X-ray fluorescence microscopy (XFM) provides powerful opportunities to advance bio-nanotechnology by assessing the compositional distribution of nanomaterials in thick biological specimens, such as hydrated whole cells or a few tens of microns thick tissue samples.^3–5^ When the incident X-ray has photon energy greater than elemental specific electron binding energy, a characteristic X-ray fluorescence (XRF) signal is emitted upon X-ray–sample interaction.^6^ This allows detection of biologically relevant trace metals such as manganese (Mn), iron (Fe), copper (Cu), and zinc (Zn),^6^ and/or other toxic metals such as mercury (Hg) and selenium (Se)^7,8^ to reveal their pathological significance and impacts on complex biological systems. In a typical synchrotron-based XFM setup, as shown in Fig. 1, incident X-ray photons are monochromatized and focused onto a sample. The sample raster scans across the focused beam to form two-dimensional (2D) elemental maps. Conventionally, an instrument is referred as a microprobe when the focused beam size is 1 μm or less, and a nanoprobe when the beam size is less than 100 nm. In addition to 2D mapping, XRF computed tomography is also exploited to investigate the elemental distribution and co-localization in a 3D space without physically sectioning the samples. A tomographic analysis is often accomplished by collecting a series of 2D XRF maps (i.e. projections) as a specimen rotates along a vertical axis (indicated in Fig. 1), followed by data reconstruction to extract the volumetric information computationally.^9^ In biological systems, tomography often helps determine whether specific element distribution features are within or on the surface of cells or cellular compartments. For example, XRF tomography measurements on a whole diatom (*Cyclotella meneghiniana*) with 400-nm spatial resolution over a 15-μm field of view showed Mn and Fe rings in the cell wall and Zn inside organelles.^10^ Nanoscale XRF tomography is equally possible, and heterogenous Zn distribution at the polar ends of the cells and uniform distribution of calcium (Ca) were found in *Escherichia coli* cells using XRF tomography with a sub-15-nm probe, without staining or sectioning the cells.^11^ These insights accomplished by XRF tomography would not be possible solely relying on 2D XRF data.

While there have been significant developments in both instrumentation and data reconstruction for scanning probe XRF tomography, this characterization technique often encounters practical challenges caused by the sample itself. During tomographic data collection, the angular coverage is limited by geometric constraints because the sample support material or sample features can obstruct/block or intrude into the field of view of a given region of interest (ROI) from which data are collected. While this issue can be avoided by scanning a large enough region to cover all surrounding materials throughout the sample rotation, in practice, this is often not possible. Currently, it is not feasible to perform point-by-point measurements over an area of hundreds of microns on a specimen when using scanning probes at a nanometer scale because of the prohibitive amount of time required for serial data collection. Even if sufficient data collection time is granted, 2D XRF projections at grazing incident angles are challenging on a 2D-like specimen, such as thin and laterally extended tissue, due to the convoluted XRF signals from a large beam–sample interaction volume at low grazing angles when the specimen is highly tilted. To overcome these challenges, we have developed a workflow that expands the tomographic rotation window, particularly for nanoscale XRF tomography. We demonstrate this workflow using a specimen containing HeLa cells exposed to Fe_3_O_4_/TiO_2_ nanocomposites.

The workflow involves three major steps: (1) identifying the location coordinates of probable ROIs and assessing their surrounding features in micron- or millimeter-sized vicinity using 2D and 3D XRF data acquired at a microprobe-XRF (µ-XRF) beamline; (2) utilizing a dual-beam scanning electron microscope (SEM) with focused ion beam (FIB) milling capabilities^12^ to create clear beam paths by removing materials surrounding the ROIs, which can interfere with data collection; and (3) characterization of the ROIs by nanoscale XRF tomography. The extended-area XRF analysis with a microprobe allows surveying a large population of cells to evaluate sample heterogeneity from which we can identify ROIs for further analysis at a subcellular level. Sample knowledge and elemental distribution learned from the 2D/3D µ-XRF results are employed to predict optimal sample orientations in the nanoprobe instrument used in Step (3). In conjunction with FIB sample manipulation, the ROIs can be precisely located with minimal ambiguity regardless of the specimen orientation. This workflow provides a crucial protocol to reveal the biological impact of nanomaterials at different length scales in complex biological systems, allowing deep insights into the nanoscale interaction between nanocomposites and cells as well as statistical measurements of the spatial distribution of nanocomposites within cells on the microscale.

## Results and discussion

With the flexible setup provided in most synchrotron facilities, each XRF beamline offers a unique combination of operation modes with differences in incident energy range, probe size, photon flux, and field of view. These operation modes allow us to detect and quantify trace elemental and chemical distributions at different spatial resolutions and length scales.^6,13^ When the spatial resolution increases, the field of view is limited. X-ray beamlines are selected considering the type of samples, including the expected feature size, elements of interest, and the concentration of these elements. For all beamlines used herein, the specimen of interest was mounted on a 500-nm-thick silicon nitride (SiN*_x_*) transmission window supported by a 200-µm-thick silicon (Si) frame and then introduced to the selected instrument. In this work, we are interested in assessing the submicron distribution of non-targeted Fe_3_O_4_/TiO_2_ nanocomposites taken up by the cells during cell division.^14^ Incident photon energy of 10 keV was used for all the XRF measurements to excite the characteristic XRF signals from the nanocomposites and the native cellular elements, including, e.g., sulfur (S), phosphorous (P), and Zn.

**Fig. 2:**
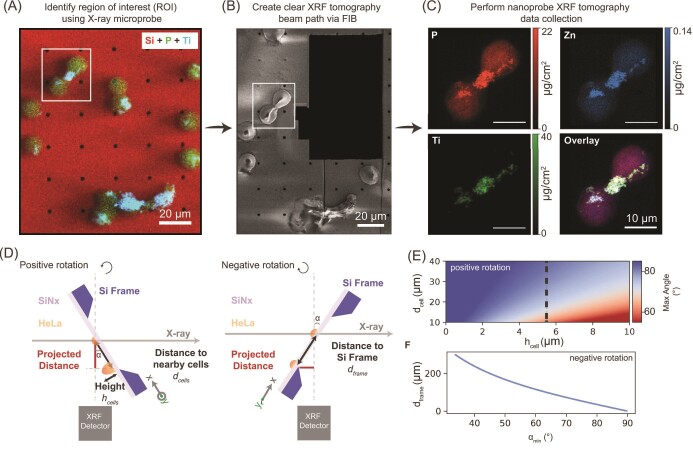
Overview of proposed workflow and geometric constraints in nanoscale XRF tomography. Multiscale X-ray characterization workflow consisting of (A) microprobe-XRF (μ-XRF) maps, overlaying data with Si, P, and Ti shown as red, green, and cyan color, respectively; (B) secondary electron image showing the sample after FIB manipulation; and (C) Bionanoprobe (BNP) mapping of selected HeLa cell pairs containing Fe3O4/TiO2 nanoparticles. (D) Schematic diagram illustrating geometric constraints in both positive and negative sample rotations. (E) Geometric prediction of the effect of nearby feature distance and height on achievable tomography angular coverage. (F) Minimum rotation is required in a negative rotation to obtain a clear beam path.

Our novel sample processing workflow is shown in Fig. 2A–C. This approach utilizes multiscale XRF data collection with additional sample processing between imaging at different beamlines. The first stage of the process is µ-XRF mapping with a beam spot size of 600 nm, allowing data collection over a mm-sized area. A subset of the data is shown in Fig. 2A. The overlay µ-XRF data, with Si, P, and Ti shown as red, green, and cyan colors, respectively, provide an initial assessment of nanocomposite distribution in HeLa cells. Congruent with the flow cytometry findings generated with the same samples,^14^ nanoparticles are the most abundant in cells undergoing cell division. We captured eight cells in their division process, two of which had a high nanoparticle accumulation in their cytokinetic bridges, as shown in Supplementary material Fig. S1. The variation of nanoparticles distribution likely indicates these cell pairs were at different cellular division stages. In Fig. 2A, one of the cell pairs with an enriched nanoparticle accumulation in the cytokinetic bridge, as highlighted in a white box, was selected as the ROI for demonstrating the workflow in this work. To deepen our understanding of the interaction between nanocomposite and subcellular structures, we implemented the intermediate sample preparation step to facilitate subsequent nanoscale measurements. Here, we used an FIB instrument with a gallium (Ga) ion source to create a clear XRF tomography beam path. A focused Ga beam with 30 kV and 10 pA operated at room temperature was used to remove the nearby HeLa cells distributed along the X-ray beam path. A secondary electron image of the specimen after FIB operation is shown in Fig. 2B. Lastly, Fig. 2C displays one projection of the nanoscale tomography dataset of the ROI collected at the Bionanoprobe (BNP)^15^ with a pixel size of 100 nm and a scan area of 20×30 µm^2^. From the BNP data, one can see that Ti is not uniformly distributed in the cytokinetic bridge, and the Ti-rich “tails” extending from the cytokinetic bridge into cell bodies, possibly following the remnant spindle components. While additional work will be necessary to establish details of the structure generated by interactions between cellular proteins and nanocomposites, it has been established that this type of nanocomposites generates complexes with baculoviral inhibitors of apoptosis repeat-containing 5 (BIRC5) and actin.^14^ Both of these proteins participate in mitotic spindle structure.

To understand the geometry constraints during BNP tomography data collection, two schematic diagrams are provided in Fig. 2D, representing a positive and a negative sample rotation, respectively. In all these measurements, an XRF detector is affixed at 90° with respect to the incident X-ray beam path. The 0-degree sample position is when the sample normal is parallel to the incident X-ray beam. From the 0-degree position, the rotation stage can rotate 90° in both positive and negative directions. Features and their spacing are projected at rotation angle $\alpha \ $along the scanning axis during rotation. Larger $\alpha $ results in smaller spacing (${{\rm{d}}_{{\rm{cell}}}}\cos \alpha\! $, where d_cell_ is the distance between nearby cells), requiring a finer probe and step size to distinguish closely packed features. When the sample rotates to a small positive angle, the emitted XRF signal can be blocked or attenuated by the nearby cells, considered as the shadowing effect, which practically limits the angular coverage of XRF tomography data collection. The degree of shadowing and achievable tomography angular coverage can be estimated by cell height and the distance between the cells, as shown in Fig. 2E. The 2D heatmap (Fig. 2E) is computed using the height and distance of features/cells on the x- and y-axes, ranging from 0 to 10 and 10 to 40 µm, respectively. From red to blue, the gradient color axis reflects the achievable tomography angular coverage, $90^{\circ} - {\tan ^{ - 1}}( {\frac{{height}}{{distance}}} )$. Fig. 2E suggests an XRF signal can be affected significantly when cells are thick and densely packed, where a reduction of more than 20° in angular coverage is predicted compared to a specimen with thin and loosely packed features. The dashed line in Fig. 2E indicates the approximate thickness of HeLa cells of 5.5 µm, where a 70° angular coverage in the positive rotation is possible when cell spacing is larger than 15 µm. Note that we consider the XRF detector as a point detector located on the dotted line in Fig. 2D with a narrow solid angle. An increase in solid angle in real measurements can reduce the shadowing effect. With these geometric predictions and initial µ-XRF evaluation, we utilized an FIB to remove the nearby HeLa cells and enlarged the angular coverage up to 20° for subsequent nanoscale tomography.

The sample substrate is the main limiting factor in a negative rotation. As illustrated in Fig. 2D, the Si frame (200 µm thick), i.e. the supporting substrate for the 500-nm SiN*_x_* membrane, will block the XRF signal from reaching the detector until the length of the projected path (in red) is larger than the thickness of the Si frame. Figure 2F computes the minimum rotation angle *⍺_min_* required to yield a non-blocking XRF signal in a negative sample rotation. The critical rotation angle is calculated using the prediction of ${\alpha _{min}} = {\tan ^{ - 1}}(\frac{{{t_{frame}}}}{{{d_{frame}}}}\ $), where *t_frame_* is the Si frame thickness and *d_frame_* is the sample-to-frame distance. When *d_frame_* is >300 µm, further away from the Si frame, a larger range of sample rotation is covered, with ${\alpha _{min}}$ smaller than 30°. On the other hand, at a small negative rotation, when the beam path of outgoing fluorescence in an Si matrix is short, the detection of XRF signal through an SiN*x* film is possible since an XRF signal is not being heavily absorbed or attenuated.

**Fig. 3:**
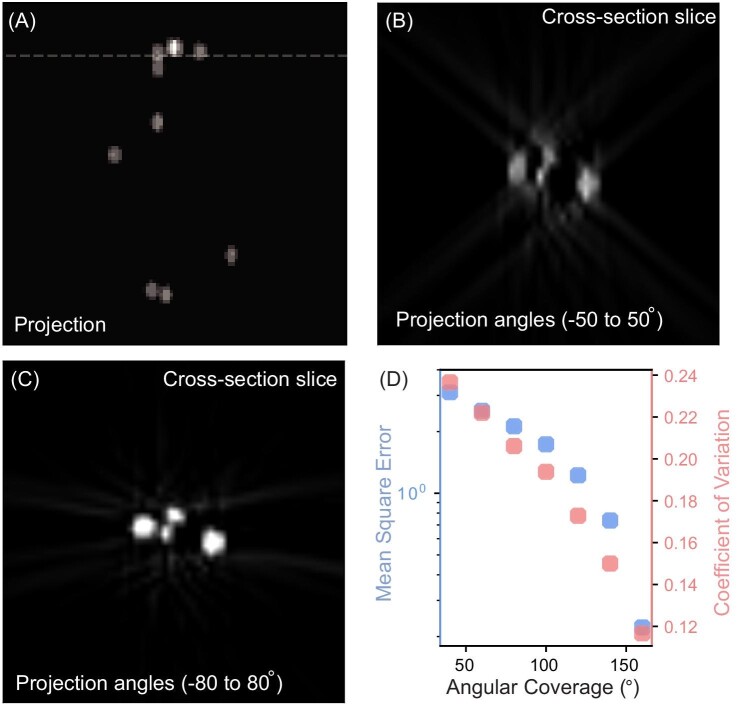
Quality of tomographic reconstruction as a function of angular coverage. (A) 2D projection of a 3D phantom with sparse particles. (B) and (C) The reconstructed results showing the cross-section information indicated by the dashed line in (A). (D) Change in mean square error (MSE) and coefficient of variation (COV), between reconstruction and phantom ground truth, as a function of angular projection coverage.

Recognition of the geometric limitations of our specimen leads us to incorporate FIB sample manipulation to expand angular coverages in nanoscale tomography. This allows for better XRF tomographic reconstruction. A decrease in both mean square error (MSE) and coefficient of variation (COV) is observed when increasing angular coverage, which is demonstrated with a phantom as shown in Fig. 3. To mimic the distribution of nanocomposites, a 3D simulated volume, known as a phantom, with sparse particles, was created using a simulation library implemented in MATLAB,^16^ whose 2D projection at 0 degree is shown in Fig. 3A. Stacking multiple 2D projections at different angles leads to the creation of a 3D data matrix whose axes are x-, y-, and rotation. This data matrix can be sliced along the horizontal axis to visualize the evolution of features during rotation, which is known as a sinogram. A tomographic reconstruction algorithm relies on the sliced information (horizontal slice indicated in Fig. 3A) to compute the cross-section information with results shown in Fig. 3B and C. Although a simultaneous iterative reconstruction technique (SIRT) algorithm was used,^17^ missing-wedge artifacts and fuzzy edges are present when projections were limited in the range of –50 to 50° (an angular coverage of 100°) as shown in Fig. 3B. In contrast, particles with sharp edges were reconstructed when the same number of projections covers –80° to 80°, illustrated in Fig. 3C. The quality of tomographic reconstruction as a function of angular coverage was examined using MSE and COV. MSE and COV were computed and compared against the phantom ground truth (*i.e.* the true values in the 3D data matrix). In Fig. 3D, a gradual decrease of MSE and COV is observed and expected as angular coverage increases. This also suggests a better reconstruction with up to 20% reduction in MSE and COV is possible when the angular coverage is expanded by 10°–20° during data collection.

**Fig. 4:**
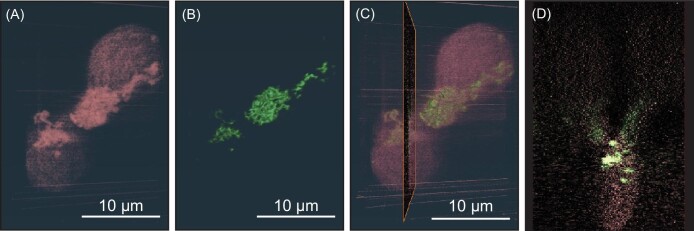
Visualization of tomography reconstruction results for P and Ti elemental channels. (A) and (B) are the 3D rendered data for P and Ti, respectively. The overlay image for these two elemental data is shown in (C). (D) displays an orthogonal slice indicated in (C). Comparable and complementary elemental analysis using the PicoProbe is shown in Figure S2.

Finally, the specimen altered by FIB, shown in Fig. 2B, was subjected to analysis at the BNP. Nanoscale tomography was performed on the single pair of cells. Reconstruction and visualization were done with an SIRT reconstruction algorithm from the ASTRA toolbox^18^ and Avizo software, respectively. Ultimately, this resulted in the 3D volume-rendered data shown in Fig. 4. Figure 4 shows the data from two elemental channels, P and Ti, and one of the orthogonal slices. Comparable and complementary elemental analysis using the high-resolution PicoProbe (analytical transmission electron microscope) was carried out post X-ray measurement as shown in Supplementary material Fig. S2. Although the nanocomposites are non-labeled, their uptake by cells is significant, as observed in previous studies with TiO_2_ or TiO_2_ shell nanoparticles.^19^ Ti and the most intense portion of the P signal have a similar distribution pattern because of the presence of phosphate in nanocomposite coating. At the same time, P is present throughout the cell body with a slightly higher concentration in the cell center, probably corresponding to newly generated nuclei of the daughter cells. In this reconstruction, fine features are observed in Ti reconstruction, which could not be resolved using the BNP tomography directly without the current workflow.

The proposed workflow was successfully implemented with an improved tomographic reconstruction for multiscale analysis of HeLa cells exposed to Fe_3_O_4_/TiO_2_ nanocomposites. Incorporating sample preparation steps with micrometer or nanoscale manipulability is crucial in the proposed workflow. However, care must be taken in sample preparation via FIB as Ga deposition was found in the surrounding area after FIB using 10 pA, the lowest beam current in the system (shown in Supplementary material Fig. S3). When the sample was FIB milled using a current ranging from 10 to 150 pA, Ga was found up to 20 µm away from the target milling area, as shown in Supplementary material Fig. S4. When using an incident X-ray beam energy of 10 keV, Ga composition was not measured, because photon energy >10.37 keV is required to remove K-shell electrons^20^ and the L emission lines are below the lowest detectable energy. Although the presence of Ga did not directly interfere with the elements of interest in the study of HeLa cells, it can act as a signal absorbing layer that reduces trace elemental XRF signal reaching the XRF detector. Extra steps to clean the sample surface or perform FIB milling using other ion sources such as oxygen^21^ and xenon^22^ can be considered to minimize or avoid Ga deposition. Monte Carlo simulation is often helpful for predicting the interaction volume, penetration depth, and beam trajectories with beam incidents on samples at various angles, energy, and beam current.^22,23^ Simulation together with optimization experimentally is able to provide good control and avoid sample damage during the FIB step.

## Materials and methods

### Biological specimen: HeLa cells treated by Fe_3_O_4_/TiO_2_ nanocomposites

The same samples used in the prior work^14^ were imaged using the novel workflow detailed in this work. The nanocomposites made of an Fe_3_O_4_ core and a TiO_2_ shell were synthesized following the same preparation procedure as in the earlier studies.^4,14,24^ Fe_3_O_4_ nanoparticles were synthesized by adding FeCl_2_ and FeCl_3_ in 24-mM citric acid. The mixture was stirred at room temperature and aged at 70°C, yielding Fe_3_O_4_ nanoparticles 1.5–3 nm in size. These Fe_3_O_4_ nanoparticles were covered by a TiO_2_ shell by adding chilled TiCl_4_ solution (−20°C) dropwise to a diluted Fe_3_O_4_ colloid. All chemicals were obtained from Sigma-Aldrich. Nanocomposite colloid was mixed with dopamine as a powder to generate a partial nanoparticle surface covering. Final nanocomposites were dialyzed in 10 mM Na_3_PO_4_ and 40 mM NaCl buffer with pH ∼ 4.5 to remove excess dopamine molecules and the remaining Ti and Fe ions. The final Ti and Fe concentrations in the nanocomposite suspension were measured after dialysis using an X Series II inductively coupled plasma mass spectrometer (ICP-MS) from Thermo Fisher Scientific. Together with nanoparticle sizing data from transmission electron microscopy, these ICP-MS measurements were used to establish nanocomposite concentrations.

A cervical cancer cell line, HeLa (CCL-2 ATCC, Manassas, VA, USA), was used in this work. The cells were propagated in Dullbecco's Modified Eagle Medium (DMEM), supplemented with 10% fetal bovine serum and 1% penicillin/streptomycin (obtained from Corning Cellgro, Thermo Fisher Scientific) at 37°C and 5% CO_2_. The cell line was seeded to be 80% confluent at the time of nanocomposite treatment; the dialyzed nanocomposites were added as 0.1% of the volume to complete media per T25 flask, with a final concentration of TiO_2_ of 20 µg/ml.

The cells were harvested after 24-h nanocomposite treatment and 24-h post-treatment incubation. During the final hour of post-treatment incubation, 10 µM Click-iT Plus EdU (5-ethynyl-2′-deoxyuridine) (Thermo Fisher Scientific) was added to the cells. The cells were harvested by trypsinization, washed with phosphate buffered saline (PBS), and fixed with 4% neutral buffered formalin. A portion of the cells was used for flow cytometry, which established their cell cycle distribution.^14^ The remaining cells were drop-cast onto SiN*x* windows for X-ray measurements. Microporous hydrophilic SiN*x* windows (pore size 2 μm with pitch 20 μm, Norcada, Alberta, Canada) were chosen for this study. The micropore array was used for two purposes: (1) as fiducial markers used across multiple analytical platforms, including the two X-ray beamlines, the SEM/FIB system, and the PicoProbe instrument; and (2) as internal rotation calibration for X-ray tomography analysis with details provided in the supplementary materials.

### Synchrotron-based XFM characterization

Synchrotron-based XFM analysis was performed at the Advanced Photon Source (APS) of Argonne National Laboratory (ANL). The microprobe at 2-ID-E and the BNP at 9-ID-B were used to demonstrate the benefit of utilizing multiscale microscopy in the proposed workflow, as well as to evaluate the uptake of non-labeled nanocomposites at different stages of the cell cycle. The XFM probes share a similar design concept, in which collinear undulators, a monochromator, and zone plate optics were used for achieving micro- or nano-focused probes. X-ray beam with energy 10 keV was used to quantify the elemental distribution in HeLa cells. An elevated energy (10.4 keV) was chosen to assess Ga distribution after the FIB milling process. X-ray photon flux and dwell time per pixel at 2-ID-E and 9-ID-B were at the level of 10^9^ counts/s with 50- ms dwell and 3×10^9^ counts/s with 30-ms dwell, respectively. A 2D XRF image was constructed for elements of interest by combining the signal response from multiple energy dispersive detectors, and fitting the elemental peaks for spectra acquired at each pixel.^25^ The data were further quantified using an AXO thin film standard (AXO DRESDEN GmbH, Dresden, Germany). The fluorescence peak fitting and quantification were performed using the MAPS software.^26^

### Tomography data collection and reconstruction

The angular coverage for µ-XRF tomography was from –20° to 55° with 1° increment, while the range for the BNP was from –78° to –36° and –24° to 78° with 2° increment. The missing rotation in the range from −34° to −22° was due to the presence of the Si frame blocking the XRF signal. Phase correlation^27^ was used to align the 2D projections. An SIRT algorithm from the ASTRA Toolbox^18^ was used for tomographic reconstruction.

### FIB sample preparation

The sample preparation using FIB milling was performed on a Zeiss NVision 40 dual-beam SEM/FIB system at the Center of Nanoscale Materials (CNM) of ANL. The sample was placed at a working distance of 5.4 mm and tilted at 54° with respect to the electron beam. A 30-kV and 10-pA Ga beam was used to remove the nearby HeLa cells of the ROI on the specimen to clear the beam path for subsequent nanoscale tomography data collection.

### Analytical hyperspectral electron microscopy characterization

The high-resolution hyperspectral analysis (Supplementary material Fig. S2) was performed with PicoProbe, an analytical transmission electron microscope at ANL. All measurements used a beam energy of 300 keV, probe current of ∼200 pA, and probe size of ∼1 nm. The measurements were done on the same cells as used for the X-ray microprobe and nanoprobe studies and were performed after the X-ray analysis and FIB operation.

## Conclusion

Geometric constraints to scanning XRF tomography imposed by the conventional SiN*_x_* substrates and the limitations stemming from the sample materials are explained and summarized. These constraints are primarily applied to XRF tomography performed using the nanoprobes where the field of view is often limited; they motivated us to develop the proposed workflow to improve subcellular XRF tomography and bridge the gap between micrometer and nanoscale analysis. Iterative data acquisition, sample processing with advanced capabilities of FIB, and multiscale X-ray microscopy were used to establish a reliable and efficient workflow facilitating high-resolution X-ray 3D analysis. A gradual increase of details generated by increasing imaging resolution and data density allowed us to observe those sample features that were first noted by microscopic observations at the nanoscale.

## Supplementary Material

mfac025_Supplemental_Files

## Data Availability

The data underlying this article will be shared on reasonable request to the corresponding author.
