# Supplementary material for: A reliable workflow for improving nanoscale X-ray fluorescence tomographic analysis on nanoparticle-treated HeLa cells
Source: Metallomics. 2022 Jun 25;14(9):mfac025. doi: 10.1093/mtomcs/mfac025 (PMC9434635; doi:10.1093/mtomcs/mfac025)
Supplement: mfac025_Supplemental_Files [file mfac025_supplemental_files.zip › Luo_TomoWorkflow_metallomics_SI_submitted.pdf]

Supporting Information

A Reliable Workflow for Improving Nanoscale X-ray Fluorescence Tomography Analysis on Nanoparticle Treated HeLa Cells

Yanqi Luo [1], Tatjana Paunesku [2], Olga Antipova [1], Yuzi Liu [3], Nestor J.Zaluzec [4], Zichao Di [5], Gayle Woloschak [2, *], Si Chen [1, *]

[1] X-ray Science Division, Advanced Photon Source, Argonne National Laboratory, Lemont, IL 60439, USA

[2] Department of Radiation Oncology, Feinberg School of Medicine, Northwestern University, Chicago, IL 60611, USA

[3] Center for Nanoscale Materials, Argonne National Laboratory, Lemont, IL 60439, USA

[4] Photon Sciences Directorate, Argonne National Laboratory, Lemont, IL 60439, USA

[5] Mathematics and Computer Science Division, Argonne National Laboratory, Lemont, IL 60439, USA

Micropore Array as Internal Rotation Calibration

The aspect ratio (width/height) of the circular pores changes as sample rotation due to feature projection explained in Figure 2D. Therefore, the sample rotation angle can be calculated using $\alpha = \cos^{-1}(\frac{d_{projected}}{d})$ as shown in Figure S5A, where α is sample rotation, d is pore diameter, and $d_{projected}$ is the projected d. The correlation plot in Figure S5A illustrates that stage encoder readout has a good agreement with the calculated rotation angles, especially at high rotation angles. A larger deviation is observed in the window of -20° to 25°, which is likely attributed to the scenario when the change in projected length is beyond our detection limit. The evolution of projected length is plotted with respect to the sample rotation in Figure S5B. The color shaded areas in red and blue correspond to non-detectable and detectable changes, respectively. The non-detectable window, from -20° to 20°, suggested from Figure S5B, matches the region with poor correlation in Figure S5A.

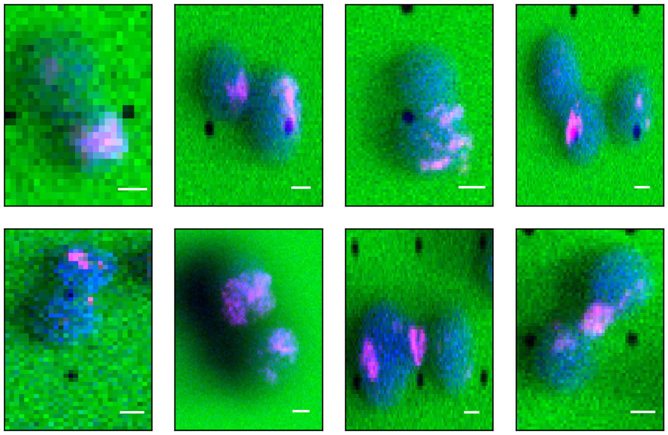

Figure S1. Dividing HeLa cells that captured during µ-XRF analysis. Overlay data of Si, P, and Ti are illustrated with green, blue, and pink colors, respectively. Length of scale bar is 5 µm.

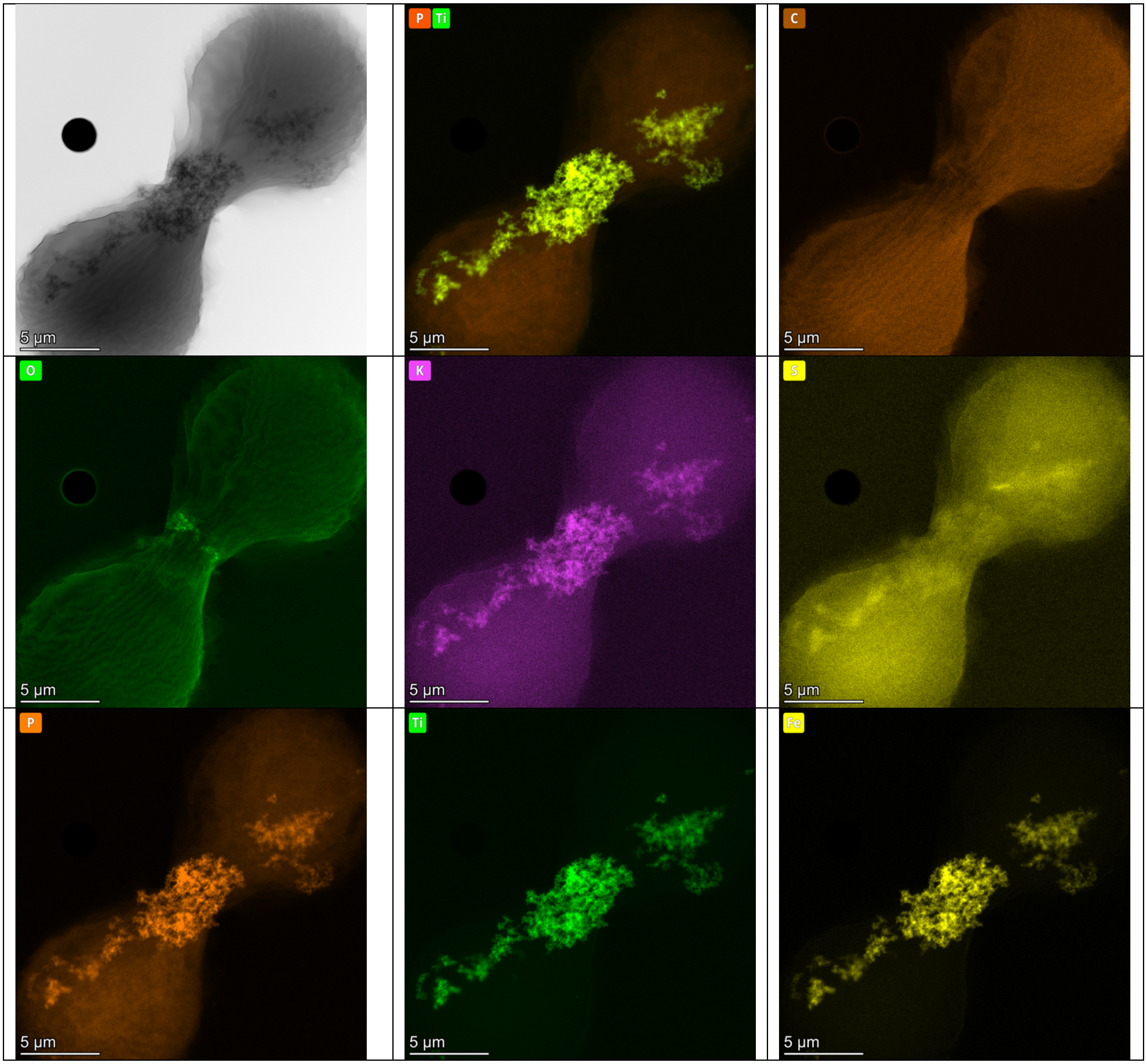

Figure S2. High-resolution hyperspectral imaging of the dividing HeLa cells collected with the analytical PicoProbe electron microscope (beam energy: 300 keV, beam diameter: ~1 nm, beam current: 200 pA, step size: ~11 nm, dwell time: 135 µsec/pixel, image size: 2Kx2K pixels) for comparison with Figures 2D and 4ABC. All colorized images are net (*i.e.*, background subtracted) hyperspectral images. The single gray-scale image is an annular dark field image of the field of view. Note the magnification in these images is slightly greater than that of Figures 2D and 4. The PicoProbe hyperspectral data were acquired after all X-ray beamline and FIB experiments were completed.

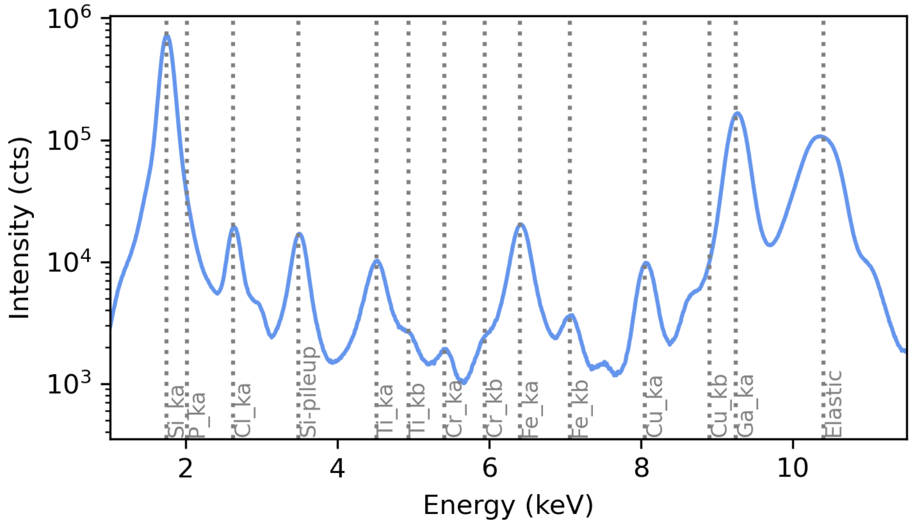

Figure S3. Integrated XRF spectra measured after FIB sample manipulation using 10.4 keV incident X-ray beam. Ga K-alpha emission line is at 9.25 keV. The XRF peaks are indicated with vertical dotted lines with their associated elements labeled to the right of the lines.

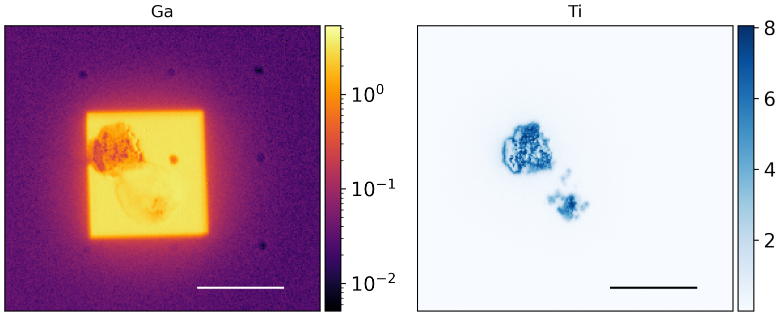

Figure S4. Microprobe-XRF (μ-XRF) measurement of a FIB milled area using 30 kV / 150 pA beam condition. The unit of the colormaps is μg/cm^2, and the length of the scale bar is 20 μm.

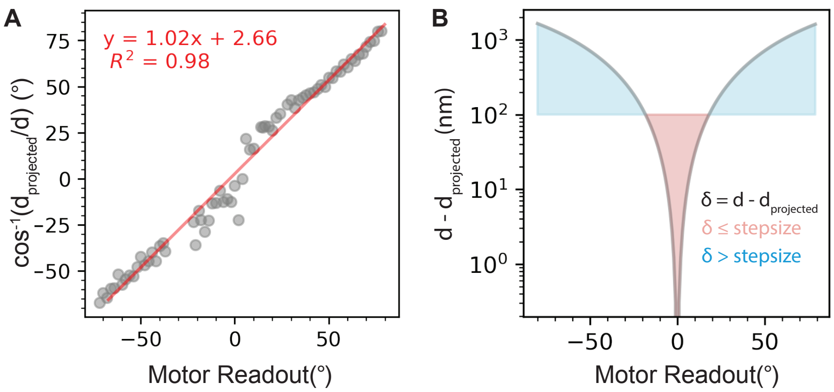

Figure S5. Potential use of micropores as the internal calibration for sample rotation. (A) Correlation plot of encoder readout vs. estimated sample rotation. (B) The change in projected length is plotted with respect to the sample rotation. The step size of data collection is 100 nm. The area between curve δ and $\delta = 100\ nm$ is highlighted in red or blue when δ is smaller or larger than the step size, respectively.
